# Exergame Intervention for Habit Formation, Lifestyle Adjustment, and Health Ownership Enhancement Among Hong Kong’s Older Adults: Multisession, Single-Group, Pre-Post Pilot Study

**DOI:** 10.2196/96571

**Published:** 2026-09-25

**Authors:** Siqiang Wang, Yi Sun, Chun Yin Chung, Yin-Leng Theng

**Affiliations:** 1 Department of Construction Management and Intelligence, The Hong Kong Polytechnic University, ZS719, 7/F, Block Z, 11 Yuk Choi Road, Hung Hom, China (Hong Kong), 852 27665565, 852 27645131; 2 Wee Kim Wee School of Communication and Information, Nanyang Technological University, Singapore, Singapore; 3 Ageing Research Institute for Society and Education, Nanyang Technological University, Singapore, Singapore

**Keywords:** exergame, exercise habit, healthy lifestyle, health ownership, motivation, older adults

## Abstract

**Background:**

Regular physical activity is critical for healthy aging, yet older adults face persistent barriers to exercise adherence. Exergames, such as video games that require physical movement, have emerged as a promising tool to engage older adults in regular exercise. However, most existing research focuses on physical and cognitive outcomes, with limited understanding of whether exergames are associated with behavioral changes, including sustained exercise habits, healthier lifestyles, and a sense of health ownership, which underpin older adults’ environmental mastery and self-management of their health.

**Objective:**

This pilot study investigated how participation in a community-based exergame program was associated with exercise habits, healthy lifestyles, and health ownership and examined the motivational factors driving longer-term adherence.

**Methods:**

This study used a single-group, pre-post pilot design. It involved 33 older adults participating in an exergame program across 2 community centers in Hong Kong. The program was meticulously designed to include 12 supervised training sessions (6 wk), followed by a 4-week free-play period to assess long-term adherence. A package of exergames, covering physical, social, and cognitive health, was selected. Each exergame session lasted 30 minutes. During this time, older adults played 3 different games, with each game taking 10 minutes. Changes in health status, well-being, physical activity level, exercise habits, healthy lifestyles, health ownership, and motivations to play exergames at 4 stages were continuously measured. We applied repeated-measures generalized linear model for health and habit outcomes, Mann-Whitney *U* to examine the relationship between motivational factors and adherence, and Spearman correlation to test links between motivation and play frequency during the free-play period.

**Results:**

The linear mixed model results suggest that participation in the exergame program was positively associated with older adults’ healthy lifestyles (mean difference 0.70, 95% CI 0.14 to 1.25, *F*_3,96_=6.52; *P*<.001, η^2^=0.17), exergame play habits (mean difference 0.50, 95% CI −0.03 to 1.03, *F*_2.77,88.57_=4.88; *P*=.004, η^2^=0.13), total physical activity metabolic equivalent of task levels (mean difference 2100.83, 95% CI −249.16 to 4450.82, *F*_2.44,78.05_=3.15; *P*=.04, η^2^=0.09), and health ownership (mean difference 0.24, 95% CI 0 to 0.48, *F*_1.61,51.44_=4.23; *P*=.03, η^2^=0.12). Posttraining adherence was high at 75.80% (25/33). Further, Spearman rank correlation analysis indicated positive associations between adhered gameplay frequency and social (coefficient=0.41; *P*=.02) and enjoyment motivations (coefficient=0.39; *P*=.02).

**Conclusions:**

This pilot study examines exergames as an innovative approach to healthy aging by encouraging sustained behavioral change. Unlike prior research that emphasized health outcomes, these findings suggest potential benefits in formulating physical activity habits, lifestyle adjustment, and health ownership. Enjoyment and social connection emerged as key motivational factors. The study indicates potential for nongovernmental organization–led community programs to foster active aging, lifestyle adjustment, and social cohesion.

## Introduction

### Exergames: A Digital Health Solution

Participating in exercise is an important strategy for older adults to achieve healthy aging. While the advantages of exercise are well recognized, many older adults struggle to adhere to regular exercise [1]. This could be due to the effort required for exercise and the perception that it can be monotonous. Offering new attractive types of exercise is crucial to encouraging older adults to commit to long-term adherence. With advancements in technology, older adults now have more opportunities for exercise, including exergames. Exergames, a blend of “exercise” and “games,” are video games that also serve as a form of physical activity. These games are designed to engage players in physical movement, often simulating activities such as dancing, sports, and fitness exercises [2]. Through gamified activities, routine activities (such as doing exercises) become more attractive and easier to adhere to.

Recent studies have demonstrated the potential of exergames in primary health care, particularly as a preventive approach to support physical and mental well-being. A Korean study reported that older adults aged above 75 years who participated in an 8-week exergame program (playing Ring Fit Adventure for 50 min, 3 times a week) showed a significant improvement in physical function, fall efficacy, depressive symptoms, and health-related quality of life [3]. A San Diego study reported that 36 sessions of training with Nintendo’s Wii gaming console (including tennis, bowling, baseball, golf, and boxing) effectively improved older participants’ depressive symptoms, mental health–related quality of life, and cognitive functioning [4]. Litz et al [5] found that exergames improve older adults’ cognition (such as memory). The intervention group showed significant improvements in dynamic balance and visuospatial memory compared to the control group. In terms of social benefits, a Singaporean study developed an exergame called “New Apple” for 319 participants aged 65 years or older (twice a week for 6 wk), identifying increased positive affect and decreased negative affect on emotional well-being among participants over time [6].

### Measurement: From Health Outcomes to Behavioral Intentions

Exergames not only contribute to individuals’ health but also support habit formation. A habit is a behavior that repeats regularly and occurs automatically, without requiring scheduling or effort [7,8]. Regular exercise activities performed consistently over time can develop habitual behavior, which becomes “volitional and mindful” as a result of learning [9]. Consequently, when people are exposed to specific contexts, such as designated locations, times, similar objects, and similar people, these contexts could activate an impulse to perform particular behaviors [10]. Exergames have been shown to effectively help people create and sustain robust exercise habits by providing virtual challenges and reward systems [11]. Such habits are often associated with stress relief, mood enhancement, social interaction, and expanded social networks, all of which contribute to the quality of life [12].

Lifestyle refers to a comprehensive pattern of living. A healthy lifestyle includes engaging in physical activity, making good nutritional choices, getting enough sleep, and avoiding harmful behaviors such as smoking and excessive alcohol consumption [13,14]. Developing such a lifestyle reflects competence and coping skills, enabling older adults to manage health conditions and maintain independence [15]. Research has shown that for older adults, developing a healthy lifestyle is linked to reduced mortality, reduced frailty, and decreased risk of chronic diseases [13].

Beyond habit and lifestyle, the study found that exergames are conducive to reinforcing “a sensitivity of the individual responsibility to care for themselves, especially at older age” [16]. This reflects mindset changes in health, from being a passive recipient of health services to proactively managing one’s own health conditions. Such reframing is often described as greater health ownership, meaning “taking control of one’s health, accepting responsibility for one’s health, and being accountable for one’s health-related outcomes” [17]. Health ownership indicates a high level of self-efficacy, whereby an individual possesses firm beliefs in their ability to effectively maintain their health by performing specific tasks or goals. This sense of responsibility may empower older adults to take charge of their health conditions and sustain healthy lifestyles.

### Motivational Factors

Older adults may recognize the importance of health and prevention, but their awareness does not always lead to behavioral changes or improved exercise habits. Exercise adherence is key, which is driven by motivation [18]. Based on an experiment using wearable equipment with 600 Turkish adults, motivation plays a vital role in stimulating behavioral repetition, which could potentially result in habit formation [7]. A cross-sectional questionnaire survey of 252 adults in the United Kingdom showed that different motivations result in distinct forms, extents, and stages of exercise participation [19]. Within this study, adults who engaged in physical exercise primarily for appearance or weight control tended to report higher external regulation, meaning that their participation was encouraged by external contingencies (eg, social pressure). The authors further noted that such external regulation was linked to lower participation levels and greater introjected regulation, such as exercising to boost self-esteem or relieve guilt [19].

Motivation could be either intrinsic or extrinsic [20]. Intrinsic factors include enjoyment and competence (such as the desire to engage in challenges and to practice skills) [21]. Extrinsic factors involve gaining body-related rewards or outcomes, including fitness and appearance [21]. The findings from 2 studies conducted by Ryan et al [21] highlighted that while extrinsic motivations often serve as the initial reasons for individuals to participate in physical exercise, intrinsic motivations (eg, enjoyment and a sense of competence) play a crucial role in maintaining long-term adherence to exercise.

To trigger intrinsic motivation, it is important to pair less desirable activities, such as conventional exercise, with more enjoyable ones [22]. Exergames provide “joyful activities such as playing a (computer-) game within an enjoyable setting” [16], which act as catalysts for continuous behaviors. A study developed the “Precious” app to increase adults’ daily physical activity and found that adding gamification elements (ie, “use of game elements in non-gaming systems to improve user experience and user engagement” [23]) to the activities could provide adults with experiences of autonomy, competence, and relatedness (eg, the freedom to chase individual goals and obtain customized feedback [24]). Intrinsic motivators, such as challenges and surprises, could lead to spontaneous behavioral change and help adults develop exercise habits. Azman Ong and Ibrahim [25] pointed out that gamified technology could change users’ behaviors because it could fulfill users’ desire for enjoyment and promote active engagement in the associated environments. Exergames can also influence individuals’ habits by promoting the learning process. Orji et al [26] developed a goal-based slow-casual game named “LunchTime” to provide education on healthier meal choices. The findings indicate that the game effectively captures users’ interest, promotes learning and reflection, and assists in developing healthy food habits.

From these perspectives, there are good reasons to believe that exergames are conducive to forming exercise habits through intrinsic and extrinsic motivations. Exercise habits contribute to the development of healthy lifestyles, which may further trigger a cognitive reframing process associated with the development of health ownership. However, this conceptual framework lacks empirical evidence, particularly for older adults. Our previous studies showed that not all older adults are familiar with digital devices and their attitudes toward smart technology vary [27]. It is also unclear which motivations play an essential role in adherence.

To address these gaps, our research aims to (1) examine the association of participation in the exergame program with the formation of exercise habits, healthy lifestyle, and health ownership among older adults and (2) to explore which motivational factors are associated with older adults’ adherence to playing exergames.

## Methods

### Inclusion and Exclusion Criteria

Inclusion criteria were being (1) aged 60 years or older, (2) able to perform simple exercises (eg, walk 360 m and move hands and feet), and (3) ambulatory without walking aids at the moment. Older adults with severe mental disorders were excluded to reduce the risk of injury. Social workers at 2 centers assisted in recruiting participants who met these criteria. We organized the introduction and trial sessions to explain the research details and familiarize participants with selected exergames. Ethical clearance was obtained before recruitment.

### Participant Characteristics

A convenience sampling strategy was used to recruit participants. Initially, the study recruited 40 older participants from 2 centers. Two participants registered for 2 centers repeatedly, 2 did not attend all training sessions in the first 6 weeks, and 1 withdrew from participation due to health reasons. Finally, 33 participants were included in the data analysis, regardless of whether they adhered to the exergame program during the free-play period. An intention-to-treat analysis was not performed, and this omission is acknowledged as a study limitation. The profiles of the participants are reported in Table 1.

**Table 1.** The profile of participants (N=33).

Characteristic	Participants
Age (y), n (%)	
60‐64	1 (3.03)
65‐69	6 (18.18)
70‐74	13 (39.40)
75‐79	7 (21.21)
80‐84	5 (15.15)
>85	1 (3.03)
Gender, n (%)	
Female	30 (90.91)
Male	3 (9.09)
Education level, n (%)	
Below primary school	4 (12.12)
Primary school	18 (54.55)
Secondary school	2 (6.06)
Postsecondary	9 (27.27)
Housing, n (%)	
Public housing (rent)	6 (18.18)
Public housing (Home Ownership Scheme)	13 (39.39)
Private housing	14 (42.43)
Motivation to participate in exergame (stage T0), mean (SD)	
Enjoyment	5.94 (1.26)
Social connection	5.60 (1.50)
Fitness	6.36 (1.02)
Competence	5.86 (1.40)
Appearance	5.62 (1.35)
Reasons to continue playing exergame (multiple choices, stage T3), n (%)	
Enjoyment of the activities	22 (66.67)
Improved physical health	19 (57.58)
Social support from friends or group participation	19 (57.58)
Accountability (eg, tracking progress)	14 (42.42)
Setting personal goals	12 (36.36)
Feeling more energetic	16 (48.48)
Stress relief	16 (48.48)
Likes taking up new challenges (ie, becoming better at something)	19 (57.58)
Likes playing new games/learning something new	19 (57.58)

### Sampling Procedures

We recruited participants from 2 centers for older adults in Hong Kong, located in urban areas with deteriorating neighborhoods and aged buildings. These areas are characterized by overcrowding, lack of public amenities, and insufficient open space, leading to more sedentary behaviors among older adults. These neighborhoods have a high proportion of older adults, mainly from low-income households with lower health literacy [28]. Poor amenities and housing conditions further hinder access to essential services and physical activities [29].

### Sample Size, Power, and Precision

As this study was designed as a single-group pilot study, a priori power analysis was not performed. Sample sizes were determined pragmatically, given feasibility constraints and the study’s exploratory design. Post hoc calculations using G*Power (version 3.1.9.7; Heinrich Heine University Düsseldorf; paired-samples *t* test, α=.05, power=0.80) indicate that the study was able to detect medium-to-large effects (Cohen *d*≈0.50). Smaller effects may not have been detectable. To reflect precision, all outcome estimates are reported with 95% CIs.

### Instrumentation

We selected 4 Nintendo Switch games through a panel of interdisciplinary experts in computer science, sport science, geriatric medicine, and urban planning. These games were chosen to target 3 key health domains, namely physical, social, and cognitive. Nintendo Switch Sports and Nintendo Ring Fit Adventure were chosen for physical health. These games leverage the console’s motion sensors and controllers to encourage upper-limb movements such as arm raising, swinging, pressing, and pulling. Social health was addressed through Super Mario Party, which fosters social interactions and multiplayer experiences, encouraging cooperation and competition among players. Cognitive health was targeted with Big Brain Academy: Brain vs Brain, which includes puzzle games designed to train cognitive abilities and mental processes across 5 categories, including identifying, memorizing, analyzing, computing, and visualizing.

### Procedure

The program consisted of 2 stages: 6 weeks of training (12 sessions) plus 4 weeks of free play. The program required the participants to attend the 12 training sessions at the center to play designated exergames under the supervision of researchers and social workers. In addition, there was a 4-week post–free-play period for older adults to continue playing exergames voluntarily without supervision. During the 12 training sessions, older adults attended 2 sessions per week. Each session lasted 30 minutes to ensure that it would not negatively affect the older adults’ health. The schedule of the program was designed to balance between program efficacy and feasibility so that it would be effective in forming habits and producing sufficient benefits [30].

**Figure 1.**
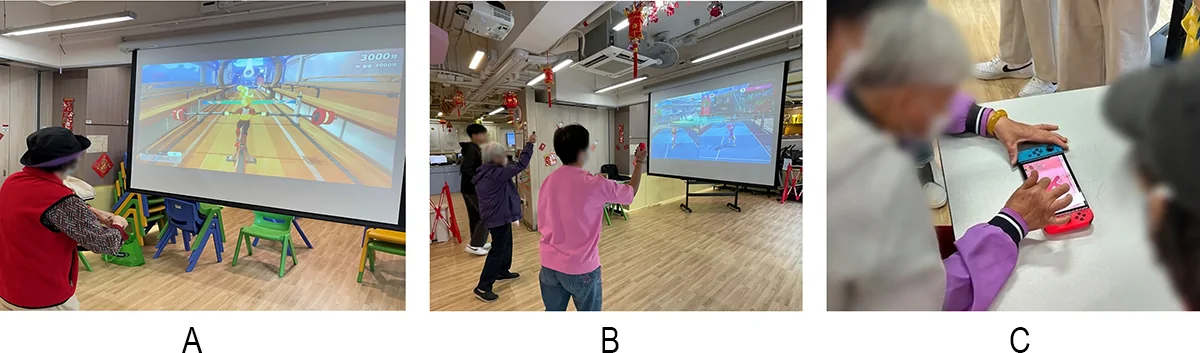
Older adults playing exergames at centers for older adults in Hong Kong. (A) Physical game; (B) social game; (C) cognitive game.

In each session, older adults played 3 selected exergames, with 10 minutes for each game (Figure 1). The social and cognitive games were played in every session, while the 2 physical games were played alternatively. Its goal was to offer a wide range of benefits, encompassing physical, social, and cognitive health improvements. After 12 sessions, participants could voluntarily continue playing exergames at community centers for 4 weeks, with no specific games or order required. The social workers helped collect attendance data during this free-play period.

### Data Collection

We conducted 4 rounds of measurements during the whole program, which were at T0 (before the training session), T1 (after 6 training sessions), T2 (after 12 training sessions), and T3 (after the 4-wk free-play period) stage. The questionnaire consists of 10 parts, including older adults’ general health, well-being, cognitive ability, exergame play habits, physical activity level (to reflect exercise habits), lifestyle, health ownership, motivation for exergame play, reasons for continuing exergame play, and their socioeconomic status (age, gender, education level, and housing types).

### Measures and Covariates

We measured general health using a 12-item Short Form Health Survey [31]. Well-being was evaluated by using the Chinese version of Keyes’s 14-item scale in 3 facets: emotional, social, and psychological well-being [32,33]. The scale has been validated as suitable for Hong Kong’s older adults [34]. Older adults’ cognitive ability was measured by the Hong Kong version of the Montreal Cognitive Assessment (HK-MoCA) [35], with higher MoCA scores indicating better cognitive ability. We used both HK-MoCA and HK-MoCA Alternate Version 1 interchangeably to minimize the “test-retest” effect. The HK-MoCA was used at T0 (week 0) and T2 (week 6), while the HK-MoCA Alternate Version 1 was used at T1 (week 3) and T3 (week 10).

We measured older adults’ health behaviors and perceptions in terms of exercise habits, healthy lifestyle, and health ownership. Older adults’ habits of playing exergames were assessed using the Self‐Report Habit Index [36]. It evaluates the extent to which people perceive their behavior as automatic and executed without conscious thought. In this study, all 12 statements were rephrased with regard to exergame play, using a 5-point Likert scale. The physical activity level was calculated based on the International Physical Activity Questionnaire, an internationally recognized scale measuring self-reported physical activity levels [37]. It was used as a proxy for sustained activity patterns and to represent exercise habits, as suggested by previous studies [38,39]. The measurement includes the physical activity levels for work, transport, and leisure. The questions to evaluate older adults’ healthy lifestyles were adapted from the Adams 5-item measurement of smoking, fruits and vegetables, exercise, sleep, and alcohol use [13].

Currently, there is no established scale measuring for health ownership. We adapted measurements from the Workplace Safety and Health Ownership Model [40], which evaluates health citizenship, health climate, and health contract. The scale was validated among 541 older adults in Hong Kong before the exergame program, with the accepted composite reliability values of 0.92 (health citizenship), 0.91 (health climate), and 0.89 (health contract), as well as average variance extracted values of 0.70 (health citizenship), 0.68 (health climate), and 0.67 (health contract).

We measured motivations for exergame play using questions adapted from Ryan et al [21], which included intrinsic factors (namely, enjoyment and competence), extrinsic factors (fitness and appearance), and motivation for social connection. This scale was validated among Chinese older adults in previous studies [41,42]. The questionnaire included 30 items, and each item was measured using a 7-point Likert scale, ranging from 1 (“corresponds not at all”) to 7 (“corresponds exactly”). The social network was assessed using the Chinese version of the Lubben Social Network Scale, which has been validated as an appropriate tool for older adults in Hong Kong [43].

### Masking

Masking was not applicable to this study, given its single-group pre-experimental design in which all participants received the same exergame intervention, and the outcome assessments were conducted using self-reported questionnaires.

### Data Diagnostics

The final dataset contained no missing data. Criteria for excluding participants after data collection included duplicate registration, nonattendance at training sessions, and withdrawal due to health reasons. No data transformations were applied. Statistical outliers were examined using box plots and were retained, given the small sample size of this pilot study.

### Quality of Measurements

To ensure the reliability and validity of the measurements, participants at both centers were provided with an introductory briefing and a trial session prior to data collection. During each gameplay period, a research assistant was present to offer assistance in the event of any difficulties. Furthermore, augmentative and alternative communication strategies were employed to enhance the accessibility of the questionnaires.

### Analytic Strategy

We performed the general linear model (repeated measures) procedure to determine whether there was a significant improvement in the general health, life satisfaction, well-being, cognitive ability, habits of playing exergames, physical activity, lifestyle, and health ownership of older adults. In addition, the Mann-Whitney *U* test was performed to examine whether and what motivations affect people’s adherence to playing exergames during the free-play period. We also conducted the Spearman rank correlation analysis to test whether older adults’ motivation for exergame play was related to their frequency of playing exergames during the free-play period. All the statistical analyses were conducted in IBM SPSS Statistics (version 29).

### Ethical Considerations

The study protocol was reviewed and approved by the Institutional Review Board of The Hong Kong Polytechnic University (reference number HSEARS20231116003). Written informed consent was obtained from all participants, who were assured that their personal data would be kept strictly confidential. All personal data were kept strictly confidential, and identifying information was removed prior to analysis. Participant records were anonymized by assigning unique codes, with names and contact details stored separately from research data. Only aggregated results were reported, ensuring that no individual could be identified from the findings. As compensation for their participation, each individual received an HK $50 (approximately US $6.40) supermarket voucher upon completion of the study. The paper does not contain any images or materials that could identify individual participants.

## Results

### The Associations Between Exergame Participation and Changes in Habits, Lifestyle, and Health Ownership

**Figure 2.**
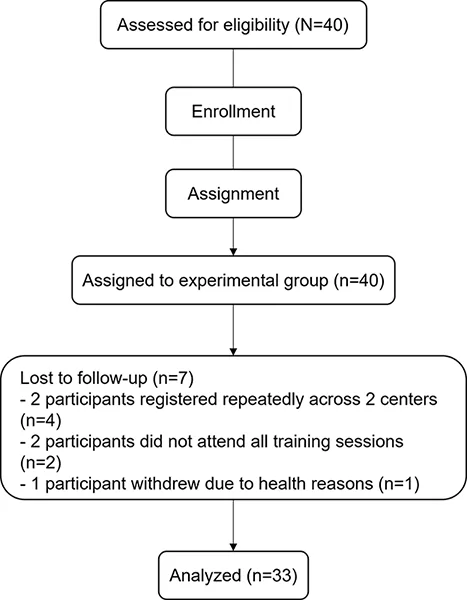
Participant recruitment flowchart.

**Figure 3.**
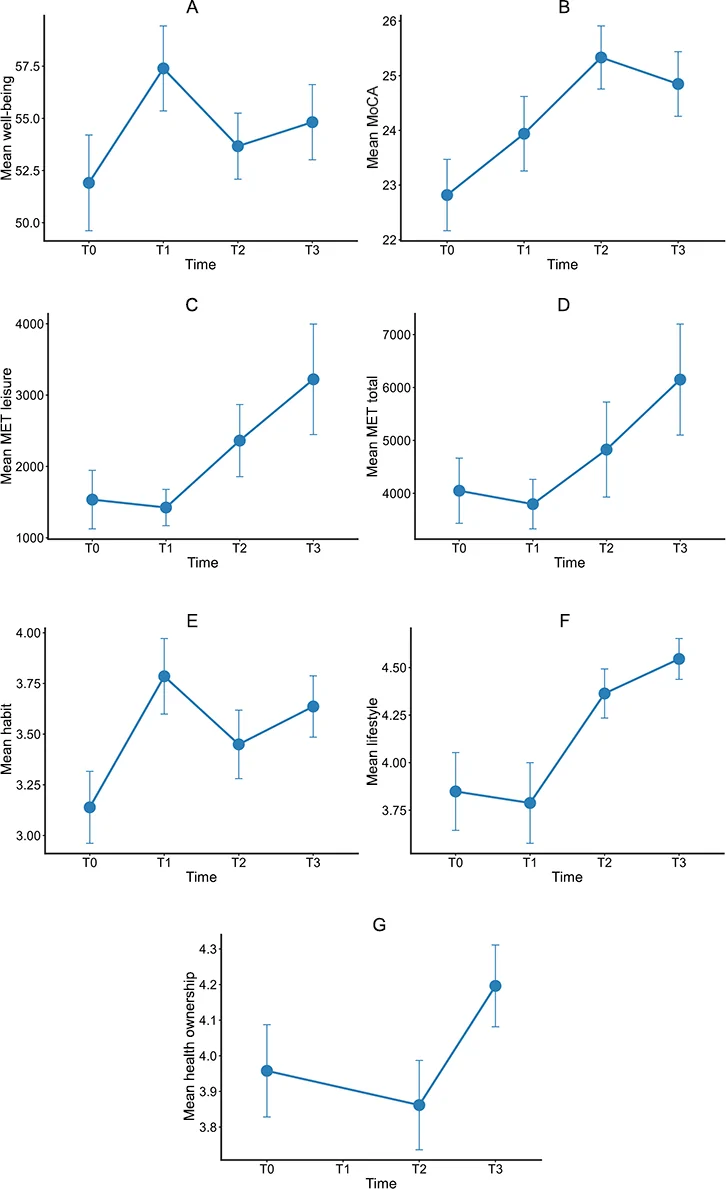
Changes in health improvement and habit formation outcomes across 4 assessment time points. (A) Well-being; (B) Montreal Cognitive Assessment (MoCA); (C) metabolic equivalent of task (MET) leisure; (D) MET total; (E) habit; (F) lifestyle; (G) health ownership.

Table 2 presents the linear mixed model results for health improvement and habit formation outcomes. Figure 2 depicts participant recruitment flowchart and Figure 3 illustrates the changes in significant health improvement and habit formation outcomes across 4 time points. The detailed post hoc comparison results are included in Multimedia Appendix 1. The results in Table 2 show that older adults’ cognitive ability improved, as indicated by MoCA scores, after participating in the exergame program (mean difference 2.03, 95% CI 0.54‐3.52, *F*_2.49,79.62_=7.86; *P*<.001, η^2^=0.20). In addition, older adults’ well-being slightly improved after participating in the exergame program (mean difference 2.91, 95% CI −2.21 to 8.03, *F*_3,96_=4.59; *P*=.005, η^2^=0.13). Moreover, the results did not report significant effects or within-group changes regarding older adults’ general health.

**Table 2.** Linear mixed model estimates of mean changes in health improvement and habit formation from baseline to week 10 (N=33).

Outcomes	Baseline (T0)		Week 3 (T1)		Week 6 (T2)		Week 10 (T3)		*F* (*df*)	*P* value	η^a^	Post hoc comparison^b^
	Mean (SD)	95% CI	Mean (SD)	95% CI	Mean (SD)	95% CI	Mean (SD)	95% CI				
Health improvement												
General health	2.55 (1.12)	2.15‐2.94	2.79 (0.96)	2.45‐3.12	2.61 (0.97)	2.26‐2.95	2.64 (0.99)	2.28‐2.99	0.69 (2.43, 77.62)	.53	0.02	—^c^
Well-being	51.9 (13.18)	47.24‐56.58	57.39 (11.70)	53.25‐61.54	53.67 (9.09)	50.44‐56.89	54.82 (10.35)	51.15‐58.49	4.59 (3, 96)	.005^d^	0.13	T0-T1
MoCA^e^	22.82 (3.75)	21.49‐24.15	23.94 (3.91)	22.55‐25.33	25.33 (3.31)	24.16‐26.51	24.85 (3.39)	23.65‐26.05	7.86 (2.49, 79.62)	<.001^f^	0.20	T0-T2, T0-T3
Habit formation												
Habit to play exergame	3.14 (1.02)	2.78‐3.50	3.79 (1.07)	3.41‐4.17	3.45 (0.97)	3.11‐3.79	3.64 (0.87)	3.33‐3.94	4.88 (2.77, 88.57)	.004^d^	0.13	T0-T1
Self-reported physical activity												
Work	1067.27 (1258.60)	620.99‐1513.55	1133.33 (1591.48)	569.02‐1697.65	1065.45 (1885.89)	396.75‐1734.16	1354.55 (1966.04)	657.42‐2051.67	0.29 (3, 96)	.84	0.01	—
Transport	1446.50 (1278.30)	993.23‐1899.77	1238.00 (1026.33)	874.08‐1601.92	1399.00 (1116.32)	1003.17‐1794.83	1574.00 (1318.90)	1106.34‐2041.66	0.55 (3, 96)	.65	0.02	—
Leisure	1535.15 (2355.39)	699.97‐2370.34	1424.24 (1460.62)	906.33‐1942.16	2362.42 (2904.28)	1332.61‐3392.24	3221.21 (4448.20)	1643.95‐4798.47	3.72 (2.38, 76.04)	.02^g^	0.10	—
Total	4048.92 (3537.19)	2794.69‐5303.16	3795.58 (2685.57)	2843.31‐4747.84	4826.88 (5158.90)	2997.61‐6656.15	6149.76 (6025.64)	4013.16‐8286.36	3.15 (2.44, 78.05)	.04^g^	0.09	—
Lifestyle	3.85 (1.18)	3.43‐4.27	3.79 (1.22)	3.36‐4.22	4.36 (0.74)	4.10‐4.63	4.55 (0.62)	4.33‐4.76	6.52 (3, 96)	<.001^f^	0.17	T0-T3, T1-T3
Health ownership^a^	3.95 (0.74)	3.69‐4.22	—	—	3.86 (0.72)	3.61‐4.12	4.20 (0.66)	3.96‐4.43	4.23 (1.61, 51.44)	.03^g^	0.12	T0-T3, T2-T3

a The older participants’ responses to health ownership were not collected at the end of week 3 (T1 stage). As health ownership indicates a high level of self-efficacy, it may not change quickly after 3 weeks of exergame training (6 sessions). Considering the length of the questionnaire and survey administration, the study did not collect participants’ responses to health ownership at the end of week 3.

b Only the significant result is reported. The Bonferroni correction is used.

c Not available.

d Significant at the .01 level.

e MoCA: Montreal Cognitive Assessment.

f Significant at the .001 level.

g Significant at the .05 level.

The results suggested a possible correlation between playing exergames and exercise habits among older adults. Specifically, the results showed a significant improvement in habits of playing exergames (mean difference 0.50, 95% CI −0.03 to 1.03, *F*_2.77,88.57_=4.88; *P*=.004, η^2^=0.13), physical activity metabolic equivalent of task (MET) level (leisure activity; mean difference 1686.06, 95% CI −219.86 to 3591.99, *F*_2.38,76.04_=3.72; *P*=.02, η^2^=0.10), and physical activity MET level (total activity; mean difference 2100.83, 95% CI −249.16 to 4450.82, *F*_2.44,78.05_=3.15; *P*=.04, η^2^=0.09) after participating in the exergame program that covered training and adherence periods. The increase in leisure-time physical activity corresponds to a MET level equivalent to brisk walking for about 1 hour per day. It shows that exergame participation is not only correlated with exergame play habits but also with people’s exercise habits (measured by physical activity MET levels). Post hoc comparisons indicated that well-being and exergame play habit scores peaked at T1 and subsequently declined, reflecting a temporal pattern of improvement followed by attenuation.

The results also report a significant improvement regarding healthy lifestyles (mean difference 0.70, 95% CI 0.14‐1.25, *F*_3,96_=6.52; *P*<.001, η^2^=0.17) after participating in the exergame program. In addition, older adults’ health ownership (mean difference 0.24, 95% CI 0‐0.48, *F*_1.61,51.44_=4.23; *P*=.03, η^2^=0.12) was significantly increased after participation in the exergame program. The exergame program not only changes older adults’ health behaviors, such as exercise habits and lifestyle, but also helps them gain a sense of mastery over their own health. Post hoc comparisons revealed that improvements in healthy lifestyle and health ownership were most evident at T3 (the end of the free-play period), suggesting that the development of these outcomes may require sustained, long-term adherence.

### Association of Motivational Factors With Exergame Adherence

In this study, motivational factors include intrinsic and extrinsic factors [21]. Extrinsic motivations involve pursuing rewards or outcomes that are unrelated to the act of exercising itself, including fitness (eg, keeping physical and mental health and having more energy) and appearance (eg, defining muscles, losing weight, and being attractive to others). Intrinsic motivation refers to fulfilling goals derived from participation in the exercise itself, including enjoyment (eg, enjoying the exergame and making me happy) and competence (eg, keeping up my skill level and obtaining new skills). In addition, the motivation for social connection is considered, such as being with others, meeting new people, and enjoying time with others.

Out of 33 participants, 25 (75.8%) continued using exergames during the free-play period (weeks 7-10). The most commonly selected reason for adherence was enjoyment of the activities (n=22, 66.67%; Table 1). Table 3 illustrates that older adults who played the exergame for social connection reasons, such as playing with friends and meeting new friends, had a higher likelihood of continuing to play the exergame after 6 weeks of training (Mann-Whitney *U*=147.00, mean difference −5.00, 95% CI −8.00 to 0, *P*=.049).

**Table 3.** Mann-Whitney *U* test results on differences in older adults’ motivation to play exergames (N=33).

Variables	Values, mean (SD)	Independent-samples Hodges-Lehman mean difference (95% CI)	Mann-Whitney *U*	Z	*P* value
Social network		0 (−4.00 to 7.00)	94.50	−0.23	.82
No (n=8)	14.88 (6.51)				
Yes (n=25)	14.12 (6.15)				
Motivation					
Enjoyment		−2.00 (−9.00 to 1.00)	138.00	1.66	.12
No (n=8)	5.79 (1.17)				
Yes (n=25)	6.27 (0.82)				
Social connection		−5.00 (−8.00 to 0)	147.00	2.00	.049^a^
No (n=8)	4.95 (1.38)				
Yes (n=25)	6.10 (0.97)				
Fitness		−2.00 (−6.00 to 0)	135.00	1.54	.15
No (n=8)	6.05 (0.87)				
Yes (n=25)	6.55 (0.51)				
Competence		−2.00 (−10.00 to 2.00)	128.00	1.19	.25
No (n=8)	5.59 (1.80)				
Yes (n=25)	6.30 (0.72)				
Appearance		−4.00 (−15.00 to 3.00)	128.50	1.20	.24
No (n=8)	4.48 (2.40)				
Yes (n=25)	5.69 (1.25)				

a Significant at the .05 level.

Table 4 reports the results of Spearman rank correlation analysis of the relationship between motivational factors and exergame play frequency during the adherence period. The results showed that motivation related to both social connection (coefficient=0.41, *P*=.02) and enjoyment (coefficient=0.39, *P*=.02) was weakly correlated with older adults’ frequency of playing exergames during the adherence period.

**Table 4.** Spearman rank correlation results for older adults’ exergame play frequency during the adherence period (N=33).

Variables	Frequency of playing exergame (monthly), coefficient (*P* value)
Social network	−0.29 (.11)
Motivation	
Enjoyment	0.39^a^ (.02)
Social connection	0.41^a^ (.02)
Fitness	0.29 (.10)
Competence	0.28 (.12)
Appearance	0.10 (.57)

a Significant at the .05 level.

## Discussion

### The Associations of Exergames Training With Habits, Healthy Lifestyles, and Health Ownership

Previous studies have shown that exergames are effective intervention tools to improve the physical and mental health of older adults [3,44]. Gamification could influence people’s perceptions by creating a virtual space that can boost their emotions and lived experiences [45]. In this study, we articulate that exergames may also be associated with behavioral and perceptual changes in terms of exercise habits, healthy lifestyle, and health ownership. Consistent, supervised exergame activities could be associated with habitual behaviors: through fixed sessions that take place twice a week, participants are willing to schedule time and devote effort to exercise regularly. This is evidenced by the increasing level of physical activities in daily life, as reported by participants.

Surprisingly, the findings indicated that the proposed exergame program had weak associations with older participants’ health status. While improvements in older adults’ well-being were observed, scores rose only at stage T1 before declining again. There are good reasons to believe that the emotional boost triggered by exergames could be temporary and nonlasting [46]. This dynamic suggests that initial gains may be driven more by short-term engagement than by the establishment of enduring behavioral routines [47]. It highlights the necessity of structured game rotation and “boost” programs (such as providing positive feedback on training outcomes) as strategies to sustain participation beyond the novelty period. Such approaches may help mitigate attrition, foster long-term adherence, and ultimately support the consolidation of healthier routines and more durable well-being outcomes. In addition, the relationship between exergame participation and older adults’ general health was not significant. One possible reason is that the selected exergames may not meet the intensity level of physical activity (ie, moderate intensity [4]). This may limit the effect of playing exergames on health promotion. In addition, the study showed that participation in the program was associated with improvements in cognitive health. Notably, the observed MoCA score improvement may be attributable to “test-retest” effects [48]. Although alternate versions were used at T1 and T3 to mitigate this effect, the absence of a control group precludes ruling out practice effects as a contributing factor to the observed cognitive gains.

Participation in an exergame program reveals a higher likelihood of formulating exercise habits among older adults. Regularly scheduled sessions encouraged participants to engage in routine physical activity, as reflected in their reported exercise patterns at stage T3. Habit formation here can be understood as a learning process that aligns with increased physical activity and psychological engagement [49]. Such exercise habits and increased physical activity levels through routine exercise may improve older adults’ physical health and psychological functions [50]. In addition, exercise habits were reported to strengthen individuals’ willingness and competence for physical activity through enhanced volition and mindfulness [8]. The latter indicates that older adults explore new life choices beyond their routine activities, leading to new norms and life goals that contribute to a more fulfilling and enriched life [51].

Participation in the program was also associated with the adoption of a healthy lifestyle. Older adults reported positive changes in 5 lifestyle dimensions that may be linked to longer life expectancy and greater adaptive capacity, which can help participants navigate challenges such as declining health status or transitions to new living environments [52]. Participation in the program was associated with a heightened sense of health ownership. The sense of health ownership enhances older adults’ personal perception of mastery and self-management of illness, especially chronic disease [53]. Health ownership is an indication of independence and autonomy in old age, which is also an important adaptation strategy [17]. Even if individuals relocate to new neighborhoods or residential care centers, having ownership over their health can help them maintain independence in unfamiliar environments [53]. This ownership fosters their ability and awareness to prioritize healthy targets, and more importantly, encourages them to take proactive measures in health management [54].

### Multifaceted Motivations for Gameplay and Adherence

Although older adults may be aware of the importance of exercise and its benefits to health, they may not always be willing to participate in exercise. This is because exercise requires effort and can be boring if there is no fun [1]. Motivation is a key driving factor in encouraging people to exercise or play exergames [55,56]. Previous studies have found that extrinsic motivation is the reason for people to participate in exercise (eg, for fitness and appearance), and intrinsic motivation (eg, for enjoyment and competence) is the reason for people to adhere to exercise [22]. The findings prove that fitness is the most common reason for older adults to participate in the exergame, which aligns with the previous study [21]. We also identify unique contributions of intrinsic and extrinsic motivation at different stages of gameplay: older adults may initially view the exergame as a playful game. Thus, enjoyment becomes an important trigger for them to participate. While playing exergames, older adults benefit from participating in activities with others, maintaining connections with friends, and meeting new friends, which helps motivate long-term adherence to exergame.

Participants reported that enjoyment and their willingness to gain competence were 2 essential motivations to participate in the 6-week training program, following fitness. This pattern differs from people’s motivations for participating in traditional exercise, in which the top 3 are fitness, appearance, and competence [21]. With intrinsic motivation (enjoyment and competence), participants may be willing to invest more resources and effort in gameplay, thereby gradually forming exercise habits [57]. Participants’ responses support the effectiveness of gamification technology in encouraging repeated exercise behavior. This is achieved by creating virtual contexts and making the setting enjoyable, which is a crucial component for triggering intrinsic motivation.

Previous studies have reported a general trend whereby adherence to exercise interventions (eg, exergames) may decline soon, even though people may show high enthusiasm at the beginning [58]. The findings suggest that older adults who have developed a strong motivation for social connection may have a higher possibility of adhering to and having a higher frequency of playing exergames in the free-play period. Such a motivation for social connection is noteworthy to mention. Research suggests that motivation for social connection contributes to both intrinsic and extrinsic motivation [59,60]. On the one hand, older adults have the opportunity to engage with others in exergames, which enhances their enjoyment and contributes to their intrinsic motivation. On the other hand, older adults may benefit from maintaining connections with their friends or meeting new ones, which contributes to extrinsic motivation.

The finding is in line with a nationwide Australian study that implemented a community-based walking program [61]. The findings reported that opportunities for social interaction are key motivators for encouraging people’s long-term participation in the program. Older adults may benefit from participating in an exercise program to meet new friends or maintain friendships with old ones [62]. Adherence to the exergame program helps expand their social networks and enhance social capital within the community. Since most exergame interventions in the existing literature are solo or competitive [63], it is essential to incorporate more elements of social interaction and engagement into exergame programs in the future.

### Limitations and Future Research

First, the findings of this study are constrained by the small sample size. One reason is that the community centers in old urban areas are severely constrained for space (eg, occupying only half a floor in an old building), which limits the potential to recruit more people at the same time. Second, the lack of a control group restricts the ability to draw robust inferences from the results. Future research should aim to recruit a larger sample size encompassing a more diverse range of socioeconomic backgrounds to better assess the impact of exergames on promoting a healthy lifestyle and health ownership among older adults. Third, most study participants were female, due to the higher rate of female engagement in activities organized by centers for older adults in Hong Kong. This imbalance made it challenging to recruit sufficient male participants. Future research should adopt random sampling across diverse community centers to enhance representativeness and generalizability. Fourth, the study relies on self-reported measures (eg, International Physical Activity Questionnaire), which introduce recall and social desirability bias. Future studies should integrate objective measurements to strengthen data validity, such as console-derived play duration or wearable-based MET tracking. In addition, the health ownership scale used in this study is adapted from the Workplace Safety and Health Ownership Model and may require further validation. Moreover, the study does not implement intention-to-treat procedures, which is a limitation, as the estimates may have been influenced by differential loss to follow-up.

### Conclusion

This pilot study suggests that exergames show promise for healthy aging, particularly in building exercise habits and fostering a healthier lifestyle. Compared to previous research, our findings indicate that the exergame program was linked only to modest gains in subjective well-being, with no significant changes in general health status. However, the training was associated with higher physical activity levels, a greater willingness to play exergames, adoption of a healthier lifestyle, and increased health ownership. These results should be interpreted with caution, given the limitations of a single-group pre-post design. Motivational factors appeared to play an essential role in sustaining adherence to the exergame program. Among these, motivations related to enjoyment and social connection (eg, companionship) were particularly important. Most participants continued playing exergames even after the supervised training ended, indicating that programs with fun activities and opportunities to connect with other participants are more likely to keep people engaged over time. Furthermore, this motivation for social connection was associated with more frequent program participation during the free-play period.

Taken together, these findings carry implications for both policy and practice. Policymakers may consider allocating resources to nongovernmental organizations for promoting exergame programs, which can be easily installed in community centers to boost participation. Singapore has already included exergames in community centers as part of health promotion. Hong Kong and other regions could consider similar initiatives to align programs with government-established, long-term lifestyle adjustment and capacity-building initiatives. Meanwhile, it is essential to include changes in lifestyle and health ownership as 2 key program outcomes, since these are vital indicators/capabilities that help older adults manage their health independently and remain proactive later in life. To maximize emotional benefits, nongovernmental organizations could refresh programs with new games and competitions to maintain continuous engagement. Collective play modes may further expand social networks and bring additional social connection benefits.

## Supplementary material

10.2196/96571 Multimedia Appendix 1 The results of post hoc comparison analysis for the health improvement and habit formation outcomes.

## Abbreviations

HK-MoCA: Hong Kong version of the Montreal Cognitive Assessment
MET: metabolic equivalent of task
MoCA: Montreal Cognitive Assessment
