# Supplementary material for: Exergame Intervention for Habit Formation, Lifestyle Adjustment, and Health Ownership Enhancement Among Hong Kong’s Older Adults: Multisession, Single-Group, Pre-Post Pilot Study
Source: JMIR Serious Games. 2026 Sep 25;14:e96571. doi: 10.2196/96571 (PMC13614622; doi:10.2196/96571)
Supplement: Supplementary file 1 [file games-v14-e96571-s001.docx]

**Appendix. The results of post-hoc comparison analysis for the health improvement and habit formation outcomes.**

(1) well-being

|  |  | Mean Difference | Std. Error | Sig.^a^ | 95% Confidence Interval for Difference^a^ | |
| --- | --- | --- | --- | --- | --- | --- |
|  |  |  |  |  | Lower Bound | Upper Bound |
| T0 | T1 | -5.485 | 1.431 | **.003**** | -9.509 | -1.461 |
|  | T2 | -1.758 | 1.597 | 1.000 | -6.248 | 2.733 |
|  | T3 | -2.909 | 1.821 | .720 | -8.031 | 2.213 |
| T1 | T2 | 3.727 | 1.376 | .064 | -0.142 | 7.596 |
|  | T3 | 2.576 | 1.464 | .528 | -1.540 | 6.692 |
| T2 | T3 | -1.152 | 1.369 | 1.000 | -5.003 | 2.700 |

a. Adjustment for multiple comparisons: ‌Bonferroni correction

* significant at 0.05 level; ** significant at 0.01 level, *** significant at 0.001 level.

(2) MOCA

|  |  | Mean Difference | Std. Error | Sig.^a^ | 95% Confidence Interval for Difference^a^ | |
| --- | --- | --- | --- | --- | --- | --- |
|  |  |  |  |  | Lower Bound | Upper Bound |
| T0 | T1 | -1.121 | 0.705 | .731 | -3.105 | 0.863 |
|  | T2 | -2.515 | 0.558 | **.000***** | -4.083 | -0.947 |
|  | T3 | -2.030 | 0.530 | **.003**** | -3.522 | -0.539 |
| T1 | T2 | -1.394 | 0.574 | .126 | -3.008 | 0.220 |
|  | T3 | -0.909 | 0.602 | .845 | -2.602 | 0.784 |
| T2 | T3 | 0.485 | 0.302 | .709 | -0.364 | 1.334 |

a. Adjustment for multiple comparisons: ‌Bonferroni correction

* significant at 0.05 level; ** significant at 0.01 level, *** significant at 0.001 level.

(3) Habit to play exergame

|  |  | Mean Difference | Std. Error | Sig.^a^ | 95% Confidence Interval for Difference^a^ | |
| --- | --- | --- | --- | --- | --- | --- |
|  |  |  |  |  | Lower Bound | Upper Bound |
| T0 | T1 | -.646 | 0.201 | **.018*** | -1.213 | -0.080 |
|  | T2 | -0.311 | 0.199 | .770 | -0.870 | 0.249 |
|  | T3 | -0.497 | 0.189 | .077 | -1.028 | 0.033 |
| T1 | T2 | 0.336 | 0.169 | .331 | -0.139 | 0.811 |
|  | T3 | 0.149 | 0.181 | 1.000 | -0.360 | 0.658 |
| T2 | T3 | -0.187 | 0.118 | .741 | -0.519 | 0.145 |

a. Adjustment for multiple comparisons: ‌Bonferroni correction

* significant at 0.05 level; ** significant at 0.01 level, *** significant at 0.001 level.

(4) Self-reported physical activity (leisure)

|  |  | Mean Difference | Std. Error | Sig.^a^ | 95% Confidence Interval for Difference^a^ | |
| --- | --- | --- | --- | --- | --- | --- |
|  |  |  |  |  | Lower Bound | Upper Bound |
| T0 | T1 | 110.909 | 379.443 | 1.000 | -956.216 | 1178.034 |
|  | T2 | -827.273 | 478.120 | .559 | -2171.911 | 517.366 |
|  | T3 | -1686.061 | 677.700 | .109 | -3591.985 | 219.864 |
| T1 | T2 | -938.182 | 559.730 | .621 | -2512.336 | 635.972 |
|  | T3 | -1796.970 | 736.804 | .123 | -3869.115 | 275.175 |
| T2 | T3 | -858.788 | 754.729 | 1.000 | -2981.345 | 1263.769 |

a. Adjustment for multiple comparisons: ‌Bonferroni correction

* significant at 0.05 level; ** significant at 0.01 level, *** significant at 0.001 level.

(5) Self-reported physical activity (total activity)

|  |  | Mean Difference | Std. Error | Sig.^a^ | 95% Confidence Interval for Difference^a^ | |
| --- | --- | --- | --- | --- | --- | --- |
|  |  |  |  |  | Lower Bound | Upper Bound |
| T0 | T1 | 253.348 | 517.081 | 1.000 | -1200.862 | 1707.559 |
|  | T2 | -777.955 | 695.797 | 1.000 | -2734.775 | 1178.865 |
|  | T3 | -2100.833 | 835.598 | .103 | -4450.821 | 249.155 |
| T1 | T2 | -1031.303 | 867.365 | 1.000 | -3470.631 | 1408.025 |
|  | T3 | -2354.182 | 945.214 | .109 | -5012.447 | 304.083 |
| T2 | T3 | -1322.879 | 1082.987 | 1.000 | -4368.611 | 1722.854 |

a. Adjustment for multiple comparisons: ‌Bonferroni correction

* significant at 0.05 level; ** significant at 0.01 level, *** significant at 0.001 level.

(6) Lifestyle

|  |  | Mean Difference | Std. Error | Sig.^a^ | 95% Confidence Interval for Difference^a^ | |
| --- | --- | --- | --- | --- | --- | --- |
|  |  |  |  |  | Lower Bound | Upper Bound |
| T0 | T1 | 0.061 | 0.217 | 1.000 | -0.551 | 0.672 |
|  | T2 | -0.515 | 0.190 | .065 | -1.050 | 0.020 |
|  | T3 | -.697 | 0.197 | **.008**** | -1.251 | -0.143 |
| T1 | T2 | -0.576 | 0.246 | .155 | -1.268 | 0.117 |
|  | T3 | -.758 | 0.234 | **.017*** | -1.417 | -0.098 |
| T2 | T3 | -0.182 | 0.147 | 1.000 | -0.596 | 0.232 |

a. Adjustment for multiple comparisons: ‌Bonferroni correction

* significant at 0.05 level; ** significant at 0.01 level, *** significant at 0.001 level.

(7) Health ownership

|  |  | Mean Difference | Std. Error | Sig.^a^ | 95% Confidence Interval for Difference^a^ | |
| --- | --- | --- | --- | --- | --- | --- |
|  |  |  |  |  | Lower Bound | Upper Bound |
| T0 | T2 | 0.096 | 0.146 | 1.000 | -0.274 | 0.466 |
|  | T3 | -.238 | 0.094 | **.048*** | -0.475 | -0.002 |
| T2 | T3 | -.335 | 0.109 | **.013*** | -0.610 | -0.060 |

a. Adjustment for multiple comparisons: ‌Bonferroni correction

* significant at 0.05 level; ** significant at 0.01 level, *** significant at 0.001 level.
